# A novel mouse model of Duchenne muscular dystrophy carrying a multi-exonic *Dmd* deletion exhibits progressive muscular dystrophy and early-onset cardiomyopathy

**DOI:** 10.1242/dmm.045369

**Published:** 2020-09-21

**Authors:** Tatianna Wai Ying Wong, Abdalla Ahmed, Grace Yang, Eleonora Maino, Sydney Steiman, Elzbieta Hyatt, Parry Chan, Kyle Lindsay, Nicole Wong, Diane Golebiowski, Joel Schneider, Paul Delgado-Olguín, Evgueni A. Ivakine, Ronald D. Cohn

**Affiliations:** 1 Program in Genetics and Genome Biology, The Hospital for Sick Children Research Institute, Toronto, ON M5G 0A4, Canada; 2 Department of Molecular Genetics, University of Toronto, Toronto, ON M5S 1A8, Canada; 3 Program in Translational Medicine, The Hospital for Sick Children Research Institute, Toronto, ON M5G 0A4, Canada; 4 Solid Biosciences, Cambridge, MA 02142, USA; 5 Department of Physiology, The University of Toronto, Toronto, ON M5S 1A8, Canada; 6 Department of Pediatrics, The Hospital for Sick Children, Toronto, ON M5G 1X8, Canada; 7 Department of Pediatrics, University of Toronto, Toronto, ON M5G 1X8, Canada

**Keywords:** DMD, Cardiomyopathy, Deletion mutation, Mouse model, Muscular dystrophy

## Abstract

Duchenne muscular dystrophy (DMD) is a life-threatening neuromuscular disease caused by the lack of dystrophin, resulting in progressive muscle wasting and locomotor dysfunctions. By adulthood, almost all patients also develop cardiomyopathy, which is the primary cause of death in DMD. Although there has been extensive effort in creating animal models to study treatment strategies for DMD, most fail to recapitulate the complete skeletal and cardiac disease manifestations that are presented in affected patients. Here, we generated a mouse model mirroring a patient deletion mutation of exons 52-54 (*Dmd Δ52-54*). The *Dmd Δ52-54* mutation led to the absence of dystrophin, resulting in progressive muscle deterioration with weakened muscle strength. Moreover, *Dmd Δ52-54* mice present with early-onset hypertrophic cardiomyopathy, which is absent in current pre-clinical dystrophin-deficient mouse models. Therefore, *Dmd Δ52-54* presents itself as an excellent pre-clinical model to evaluate the impact on skeletal and cardiac muscles for both mutation-dependent and -independent approaches.

## Abstract (precis)

**Summary:** The *Dmd Δ52-54* mouse model, which carries a deletion of *Dmd* exons 52-54, emulates Duchenne muscular dystrophy disease progression in skeletal muscle and has early onset of cardiac functional abnormalities.

## INTRODUCTION

Duchenne muscular dystrophy (DMD) is an X-linked progressive and lethal neuromuscular disease that affects one in 5000 boys (Bladen et al., 2015; Mendell and Lloyd-Puryear, 2013). The DMD disease first manifests as muscle weakness before the age of five, eventually resulting in most patients becoming wheelchair bound by 15 years old (Mavrogeni et al., 2015; Miller and Wessel, 1993). Because both skeletal and cardiac muscles are affected in DMD, patients develop cardiac and respiratory complications (Bushby et al., 2010; Judge et al., 2011). By the age of 10, 81% of patients experience cardiac involvement which includes hypertrophic cardiomyopathy (HCM), dilated cardiomyopathy (DCM), or defective cardiac conduction (Nigro et al., 1990). By adulthood, almost all DMD patients present with cardiomyopathy, where most cardiac manifestations develop into DCM at late disease stages (Mavrogeni et al., 2015; Nigro et al., 1990). While the available symptomatic management, such as ventilation, has life-prolonging effects, it also results in more patients eventually developing cardiomyopathy, which is currently the leading cause of mortality for DMD patients (Meyers and Townsend, 2019).

DMD is caused by the absence of the dystrophin protein, which is localized at the sarcolemma of the skeletal and cardiac muscles, and is an essential component of the dystrophin glycoprotein complex (DGC) (Ervasti et al., 1990). Dystrophin is comprised of key functional domains such as the actin-binding domain (ABD), cysteine-rich domain (CR), and C-terminal domain (CT), and almost 80% of the protein is composed of the largely redundant rod domain (Harper et al., 2002; Koenig et al., 1988; Nicolas et al., 2012). On the other hand, the ABD, CR, and CT domains are responsible for bridging cytosolic actin to the extracellular matrix via other DGC proteins. By recruiting the DGC components to the sarcolemma, dystrophin maintains muscle integrity during the cycles of muscle contraction and relaxation. Therefore, the absence of dystrophin expression results in the loss of DGC components, leading to DMD disease manifestation.

Dystrophin is encoded by the *DMD* gene, which at 2.3 Mb, is the largest gene in the human genome (den Dunnen et al., 1989). *DMD* is transcribed into a 14 kb mature mRNA composed of 79 exons and translated into the 427 kDa protein (Hoffman et al., 1987). The lack of dystrophin expression is due to mutations within the *DMD* gene, where 70% of the *DMD* mutation spectrum is comprised of single or multi-exonic deletions, mostly occurring within the *DMD* mutational hot spot spanning exons 2-20 and 45-55 (Bladen et al., 2015; Mendell and Lloyd-Puryear, 2013). These deletion mutations disrupt the open reading frame (ORF), producing a premature termination codon and hence preventing dystrophin expression.

Current developing therapies for DMD caused by deletion mutations revolve around restoring expression of a shorter dystrophin protein. These approaches include converting the DMD mutation into an in-frame deletion mutation found in the milder Becker muscular dystrophy (BMD), and the gene transfer of a compact dystrophin called microdystrophin (Aartsma-Rus et al., 2009; Wong and Cohn, 2017). Although these approaches have been shown to restore dystrophin expression in pre-clinical dystrophin-deficient mouse models, these small animal models have provided insight of treatment outcome mainly in skeletal muscles (Bostick et al., 2011; Hakim et al., 2017; Liu et al., 2005; Schinkel et al., 2012). These dystrophin-deficient models have a natural late onset of cardiomyopathy, and therefore are not suitable tools to study cardiac implications of these strategies.

Among the currently available DMD animal models, only a rat model (*Dmd^mdx^*) has been described to recapitulate DMD disease progression in skeletal muscle and exhibit a cardiac phenotype at 12 weeks, but it does not fully reflect cardiac function (Larcher et al., 2014). Also, *Dmd^mdx^* carries an 11 bp deletion within exon 23, which lies outside of the *DMD* mutation hot spot. Therefore, this model could only be utilized as a dystrophin-deficient model, and is less likely to be used for individualized therapeutic approaches.

Here, we generated a mouse model recapitulating the patient deletion mutation of *DMD* exons 52-54 in the mouse *Dmd* gene (*Dmd Δ52-54*). Dystrophin is absent in the *Dmd Δ52-54* mice, leading to lack of muscle fiber integrity. *Dmd Δ52-54* display dystrophic muscle histology and compromised motor strength, which is prolonged from 12 to 52 weeks. Moreover, *Dmd Δ52-54* exhibits an early onset of cardiac hypertrophy and tachycardia compared to current DMD mouse models. The *Dmd Δ52-54* deletion mutation represents 0.3% of all DMD patients (UMD-TREAT-NMD DMD database), and lies within the *Dmd* mutational hot spot. Therefore, this deletion mutation is amenable towards multiple developing therapeutic strategies for DMD that are either mutation-dependent or mutation-independent. Overall, *Dmd Δ52-54* presents as an important mouse model in DMD pre-clinical studies due to its early skeletal and cardiac phenotype, potentially revealing long-term therapeutic implications.

## RESULTS

### The generation of a *Dmd Δ52-54* mouse line using CRISPR/Cas9

**Fig. 1.**
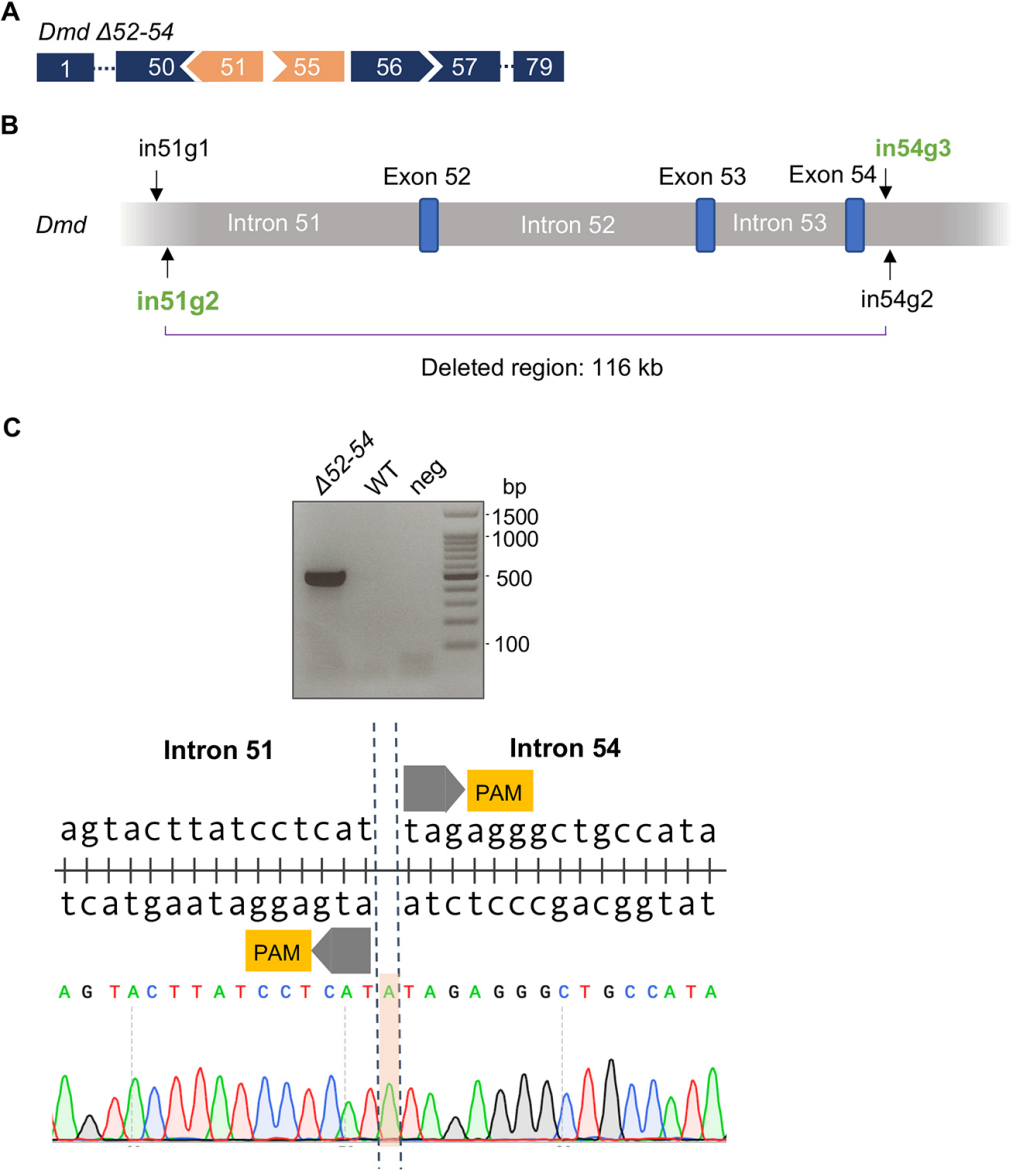
**CRISPR/Cas9-mediated generation of *Dmd Δ52-54* mice.** (A) Exon map of *Dmd Δ52-54*. As a result of the mutation, exons 51 and 55 (orange) are out of frame. (B) sgRNAs compatible with the *S. pyogenes* Cas9 system were designed to target regions within *Dmd* intron 51 (in51g1 and in51g2) and intron 54 (in54g2 and in54g3). The founder *Dmd* Δ*52-54* carried the deletion produced by in51g2 and in54g3 (green), leading to a deletion of 116 kb. (C) Genotyping with the corresponding primer sets (Table 1) detects deletion junctions present in *Dmd Δ52-54* mice. The deletion junction was confirmed using Sanger sequencing, which also detected a one base pair insertion between Cas9 cut sites (highlighted in orange). WT, wild-type control; neg, PCR negative control; PAM, protospacer adjacent motif.

The deletion of *Dmd* exons 52-54 is predicted to disrupt the ORF (Fig. 1A) leading to the lack of dystrophin expression and consequential development of a muscular dystrophy phenotype. To generate a mouse model carrying the *Dmd Δ52-54* mutation (*Dmd Δ52-54*) using CRISPR/Cas9, two sgRNAs were designed within each of intron 51 and intron 54 (Fig. 1B, Table 1). Two sgRNAs were utilized rather than one at each intronic region to enhance the frequency of deletion events. The respective sgRNAs were then delivered with *Streptococcus pyogenes* Cas9 mRNA through pronuclear microinjections, and the resultant pups were genotyped based on the presence of the intended deletion junction (Fig. 1C, Table 1). After the pronuclear injection, one out of 28 pups carried the desired *Dmd Δ52-54* mutation. The 116 kb deletion was validated through whole-genome sequencing, where non-specific events were not detected at the top ten off-target sites of each sgRNA. Sanger sequencing also revealed a 1 bp insertion between predicted Cas9 cleavage sites (Fig. 1C). In summary, we generated *Dmd Δ52-54*, the first mouse model carrying a multi-exonic deletion in the *Dmd* mutational hot spot.

### *Dmd Δ52-54* mice lack dystrophin expression and DGC recruitment to the sarcolemma

**Fig. 2.**
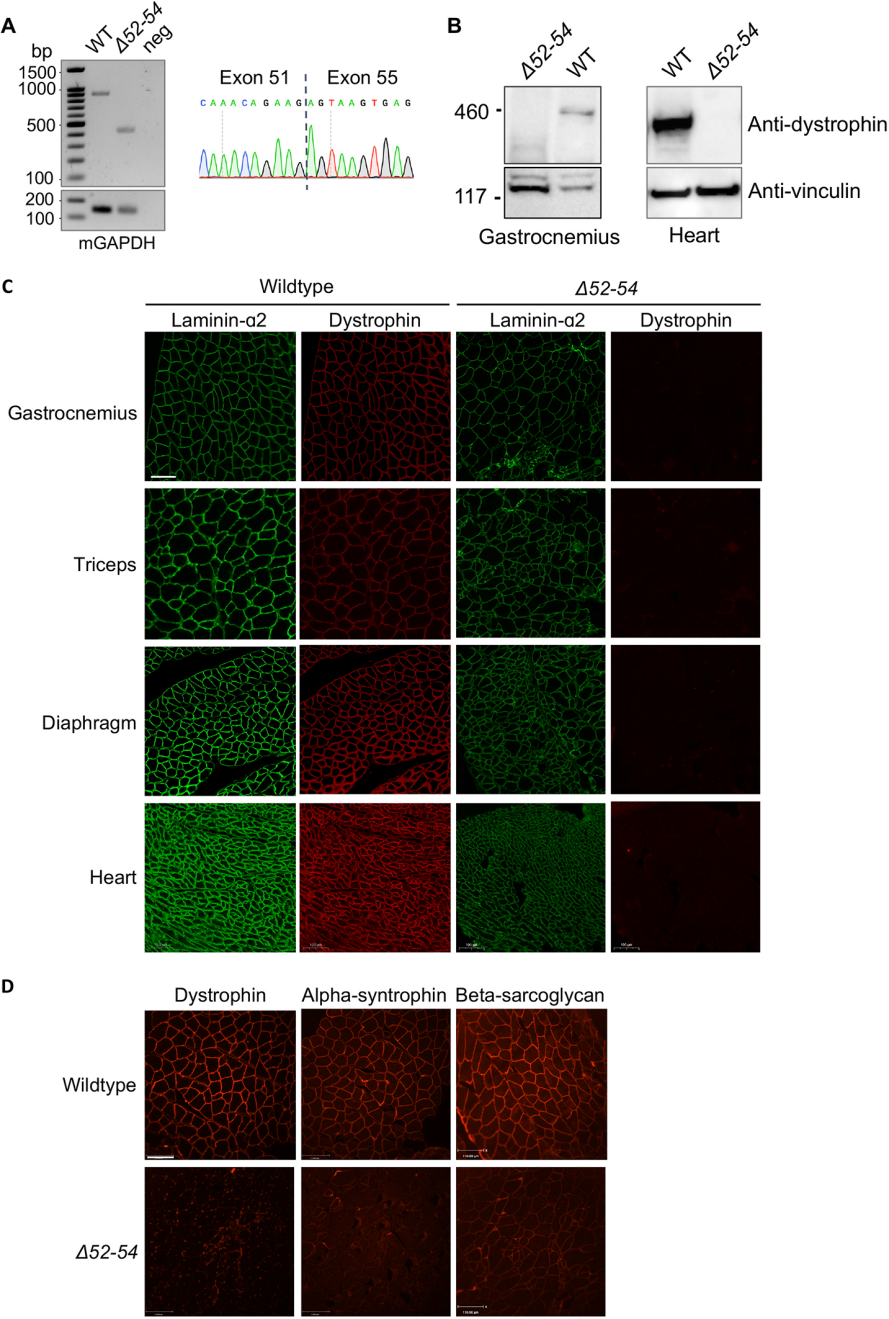
***Dmd Δ52-54* mice lack dystrophin expression.** (A) RT-PCR detection and Sanger sequencing of deletion junctions in *Dmd* transcripts in heart tissues of wild-type (WT) and *Dmd Δ52-54* (*Δ52-54*) mice. mGAPDH is shown as a control. (B) Protein isolated from gastrocnemius and heart tissues showed absence of dystrophin in *Dmd Δ52-54* mice. Anti-vinculin is used as the loading control. (C) Immunofluorescence staining detected the sarcolemma using laminin-α2 (green) and the presence of dystrophin (red) in wild-type and *Dmd Δ52-54* cross sections of the indicated tissues. Scale bar: 100 µm. (D) Immunofluorescence staining probing for dystrophin, α-syntrophin and β-sarcoglycan in gastrocnemius cross sections of WT and *Dmd Δ52-54* mice. Scale bar: 110 µm.

The mouse generation strategy using CRISPR/Cas9 was designed to leave the splice sites upstream of exon 55 unaffected. Reverse transcription PCR (RT-PCR) amplifying the region between exon 51 and exon 55 detected a deletion of 420 bp in the *Dmd Δ52-54* mice transcript expressed in heart tissues, corresponding to the deletion of *Dmd* exons 52-54 (Fig. 2A). Because the ORF is disrupted in the *Dmd Δ52-54* mutation, a premature termination codon in exon 55 was produced. Consequently, dystrophin was not expressed in *Dmd Δ52-54* mice, as observed in the gastrocnemius, triceps, diaphragm, and heart muscles (Fig. 2B,C). Due to the lack of dystrophin, DGC components such as α-syntrophin and β-sarcoglycan were not recruited to the sarcolemma (Fig. 2D).

### *Dmd Δ52-54* mice display progressive dystrophic muscle histology

**Fig. 3.**
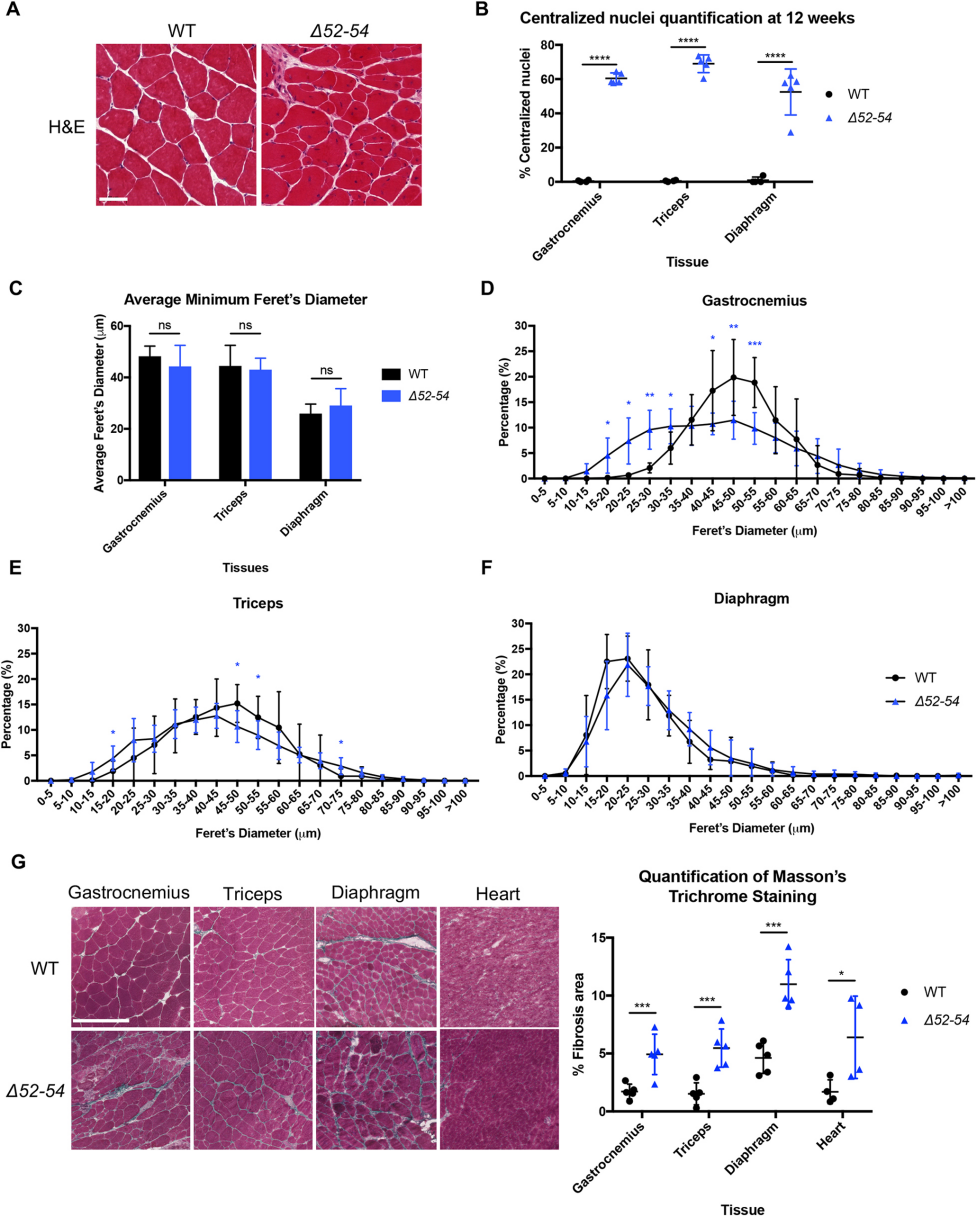
***Dmd Δ52-54* displays dystrophic histology.** (A) Hematoxylin and Eosin (H&E) staining of gastrocnemius cross sections from 12-week-old wild-type (WT) and *Dmd Δ52-54* mice. Scale bar: 50 µm. (B) Quantification of centralized nuclei in 12-week-old wild-type (*n*=4) and *Dmd Δ52-54* (*n*=5) mice. (C) Average minimum Feret's diameter and (D-F) distributions of minimum Feret's diameter were quantified using H&E cross sections from wild-type (*n*=4) and *Dmd Δ52-54* (*n*=4-5) mouse lines. (G) Masson's trichrome staining was performed on gastrocnemius, triceps, diaphragm and heart tissues of 12-week-old wild-type (*n*=4-5) and *Dmd Δ52-54* (*n*=4-5) mice, and the fibrotic area was quantified. Scale bar: 250 µm. All data are presented as the mean±s.d. Statistical analyses were performed using a Student's *t*-test. *P<0.05, **P<0.01, ***P<0.001, ****P<0.0001.

Because correct dystrophin and DGC localization is essential in myofiber integrity and muscle function, DMD patients lacking dystrophin expression experience progressive muscle deterioration. This phenotype of exacerbated muscle degeneration and regeneration is classically indicated in Hematoxylin and Eosin-stained tissue cross sections (Fig. 3A) by (1) the presence of immature myofibers containing centralized nuclei; (2) the heterogeneity in muscle fiber sizes, which illustrates the different cycles of muscle turnover (Briguet et al., 2004); and (3) the development of fibrotic tissue.

In gastrocnemius, triceps, and diaphragm from 12-week-old *Dmd Δ52-54* mice (Fig. 3A), 60%, 69% and 52% of myofibers contained centralized nuclei, respectively (Fig. 3B). While there was no difference in the average minimum Feret's diameter in skeletal muscle between *Dmd Δ52-54* and wild-type mice (Fig. 3C), *Dmd Δ52-54* myofibers in gastrocnemius and triceps were more heterogeneous and smaller in size compared to those in wild-type mice (Fig. 3D,E). Collectively, this confirmed the increased muscle turnover in the gastrocnemius and triceps of *Dmd Δ52-54* mice, but not in the diaphragm (Fig. 3F). Corresponding to the excessive muscle degeneration in *Dmd* *Δ52-54* mice compared to wild-type mice, increased fibrosis was evident in the gastrocnemius, triceps, diaphragm and heart tissues of *Dmd Δ52-54* mice by 2.9-, 3.6-, 2.4-, and 3.8-fold, respectively, further reinforcing the overall dystrophic histology (Fig. 3G).

**Fig. 4.**
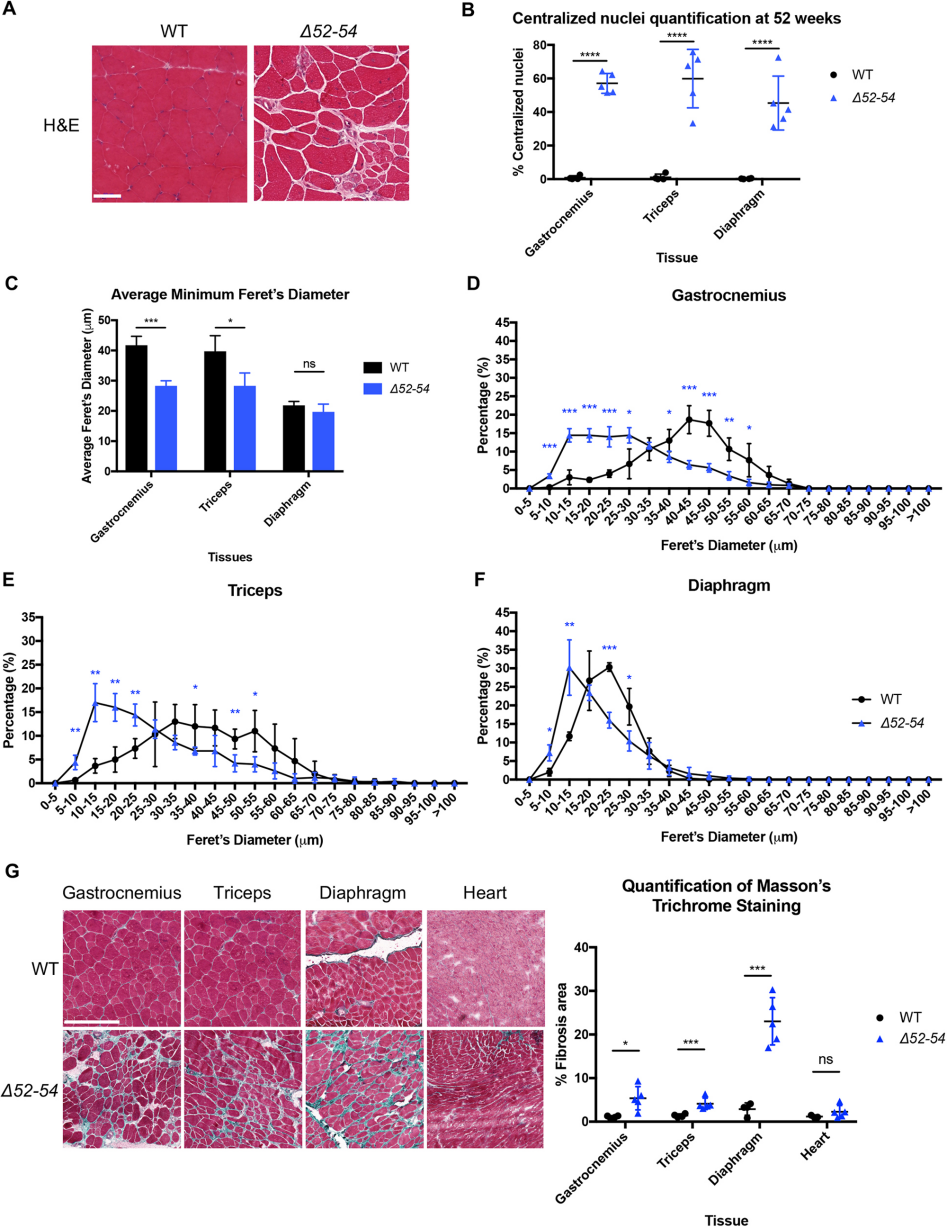
***Dmd Δ52-54* mice exhibit dystrophic histology at 52 weeks.** (A) Hematoxylin and Eosin (H&E) staining of gastrocnemius cross sections from 52-week-old wild-type (WT) and *Dmd Δ52-54* (*Δ52-54*) mice. Scale bar: 50 µm. (B) Quantification of centralized nuclei in 52-week-old wild-type (*n*=4) and *Dmd Δ52-54* (*n*=5) mice. Using H&E cross sections, (C) average minimum Feret's diameter and (D-F) distributions of minimum Feret's diameter were quantified from wild-type (*n*=3) and *Dmd Δ52-54* (*n*=5) mouse lines. (G) Masson's trichrome staining was performed on gastrocnemius, triceps, diaphragm and heart tissues of 52-week-old wild-type (*n*=3-4) and *Dmd Δ52-54* (*n*=5) mice, and the fibrotic area was quantified. Scale bar: 250 µm. All data are presented as the mean±s.d. Statistical analyses were performed using a Student's *t*-test. ns, not significant; *P<0.05; **P<0.01; ***P<0.001; ****P<0.0001.

Elevated muscle turnover was also observed in *Dmd Δ52-54* mice at 52 weeks (Fig. 4A). In the gastrocnemius, triceps and diaphragm, an average of 57%, 60%, and 45% of myofibers contained centralized nuclei, respectively (Fig. 4B). On average, there was a 28% and 32% decrease in myofiber size in the gastrocnemius and triceps, respectively, compared to wild-type mice, but such a decrease was not observed in the diaphragm (Fig. 4C). There was also a significant shift in myofiber size in the gastrocnemius, triceps and diaphragm tissues at 52 weeks, where the majority of the *Dmd Δ52-54* myofibers were smaller compared to those in wild-type (Fig. 4D-F). Regarding the diaphragm, this shift in myofiber size was only evident at an older age and not at the 12-week-old time point (Fig. 4F). There was also a 2-fold increase in fibrosis in the diaphragm at 52 weeks compared to 12-week-old *Dmd Δ52-54* mice (Fig. 4G). Taken together, the histological analyses at 12 and 52 weeks indicate that muscular dystrophy is progressive in *Dmd Δ52-54* mice, similar to that in DMD patients.

### The absence of dystrophin in *Dmd Δ52-54* mice leads to compromised muscle strength

**Fig. 5.**
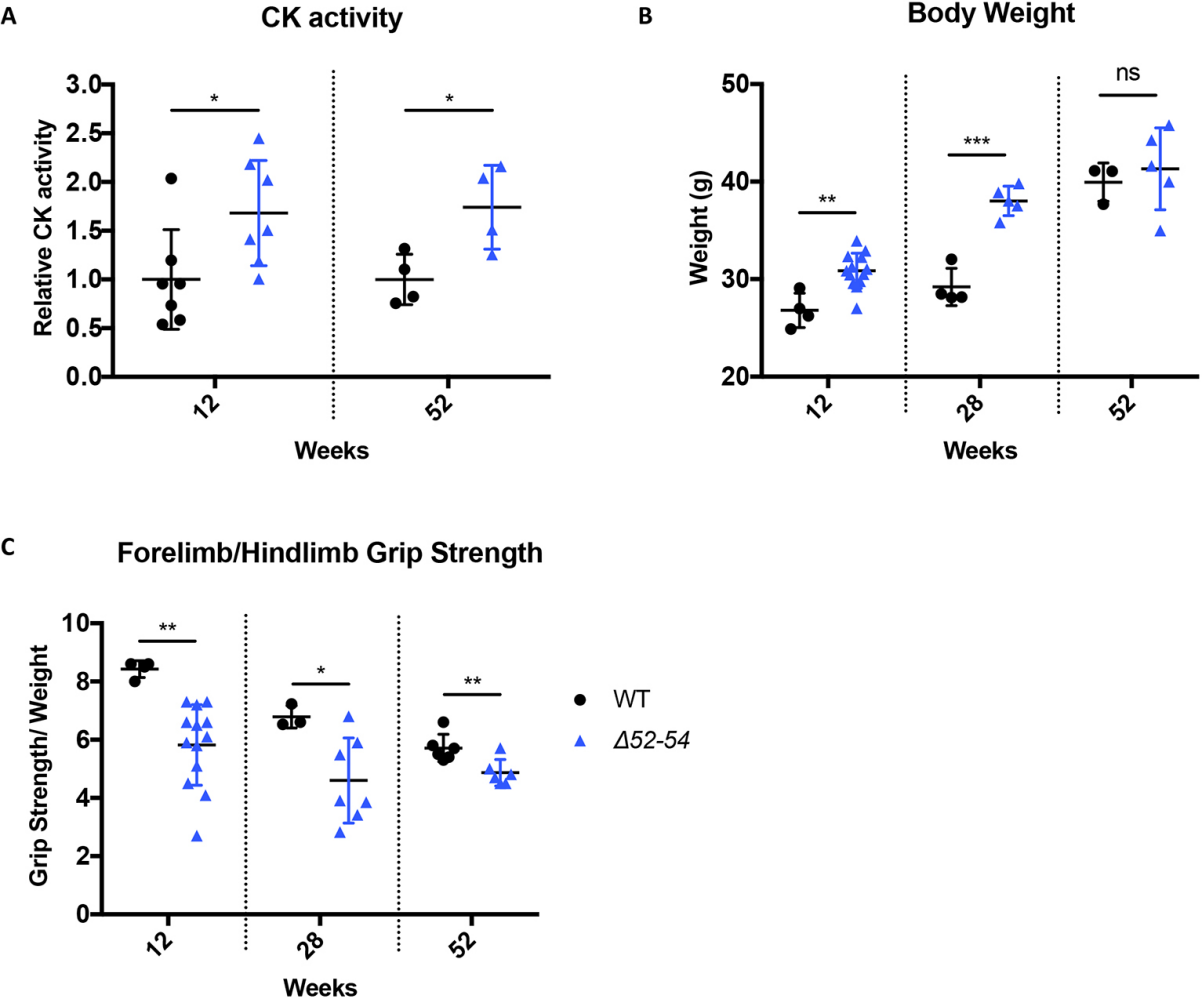
***Dmd Δ52-54* mice exhibit elevated body weight and compromised muscle strength from 12 to 52 weeks.** (A) Serum CK activity of *Dmd Δ52-54* (*Δ52-54*) mice relative to wild-type (WT) mice at 12 and 52 weeks. (B) Body weight of wild-type (*n*=4) and *Dmd Δ52-54* (*n*=13) mice at 12, 28 and 52 weeks of age. (C) Forelimb and hindlimb grip strength of wild-type (*n*=4) and *Dmd Δ52-54* (*n*=13) mice at 12, 28 and 52 weeks of age. All data are presented as the mean±s.d. Statistical analyses were performed using Student's *t*-test. ns, not significant; *P<0.05; **P<0.01; ***P<0.001; ****P<0.0001.

Owing to the loss of myofiber integrity in the dystrophin-deficient *Dmd Δ52-54* mice, there was an elevated level of serum creatine kinase of ∼1.7-fold compared to levels in wild-type mice at both 12 and 52 weeks (Fig. 5A). This further supports the presence of damaged myofibers in the *Dmd Δ52-54* mice, and hence muscle atrophy and necrosis, emulating the DMD disease. Degenerated myofibers are displaced by fat deposition and fibrosis, which leads to pseudohypertrophy (Kornegay et al., 2012), and is consistent with the heavier body weight observed for *Dmd Δ52-54* mice at 12 and 52 weeks (Fig. 5B). However, muscle histology (Figs 3A and 4A) indicated that the pseudohypertrophy observed in *Dmd Δ52-54* mice is primarily attributed to fibrotic development (Figs 3G and 4G). Because the presence of fibrosis impairs skeletal muscle function, the dystrophic *Dmd Δ52-54* mice had weakened muscle strength from 12 to 52 weeks of age, where there was a decreased grip strength by 20-30% compared to that of wild-type mice (Fig. 5C). Altogether, *Dmd Δ52-54* mice present features in accordance with a muscular dystrophy phenotype.

### *Dmd Δ52-54* mice develop cardiac hypertrophy and tachycardia

**Fig. 6.**
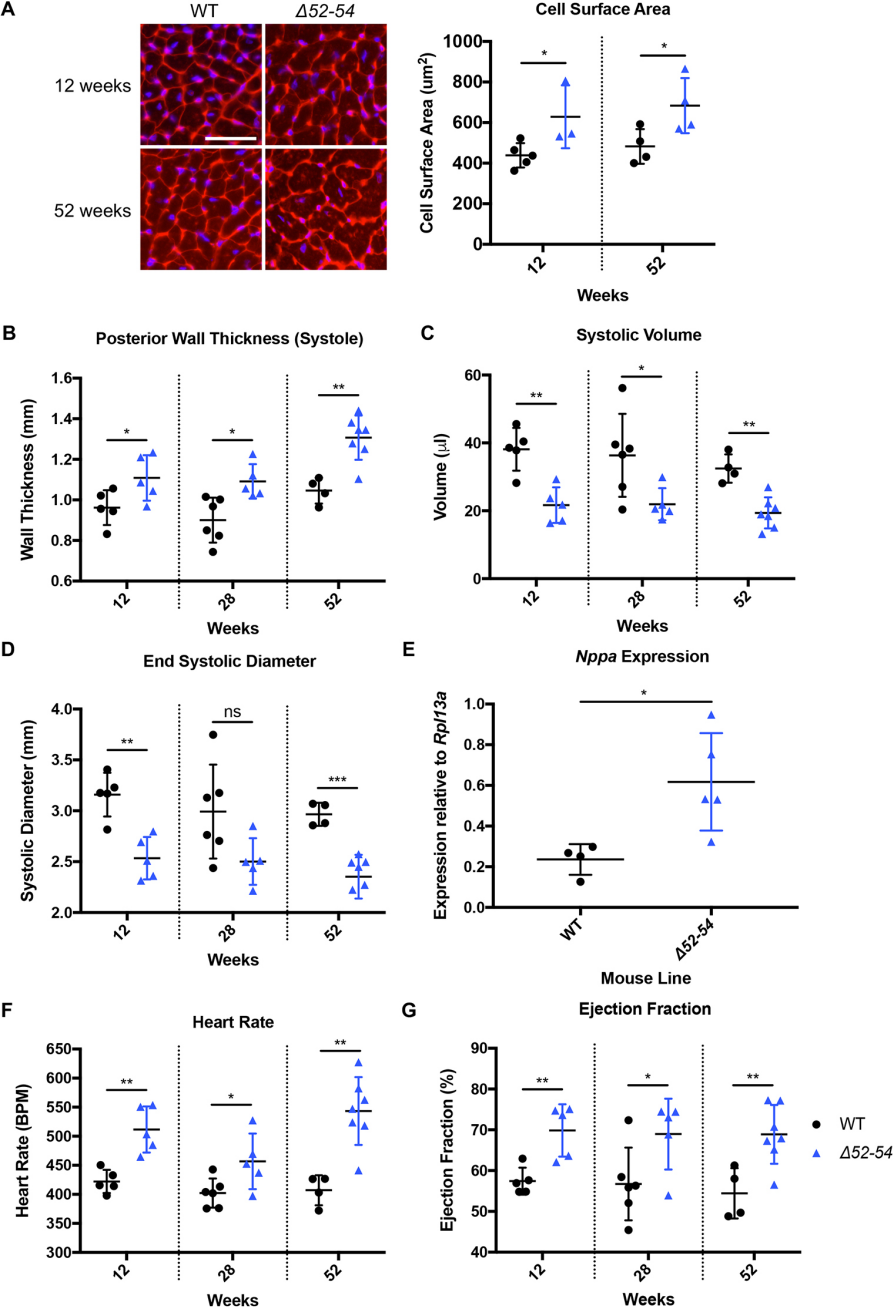
***Dmd Δ52-54*** **mice** **develop long-term persistent** **cardiac hypertrophy and tachycardia.** (A) Wheat germ agglutinin (WGA) staining of left ventricular cross sections was conducted in wild-type (WT) and *Dmd* *Δ52-54* (*Δ52-54*) mice at 12 weeks (WT, *n*=5; *Δ52-54*, *n*=3) and 52 weeks (WT, *n*=4; *Δ52-54, n=4*). Scale bar: 50 μm. (B-D) The heart in wild-type and *Dmd* *Δ52-54* mice was analyzed at 12, 28 and 52 weeks using echocardiography to determine left ventricular posterior wall thickness during systole (B), left ventricular systolic volume (C) and left ventricular end systolic diameter (D). (E) mRNA expression of *Nppa* in whole hearts of wild-type and *Δ52-54* mice at 12 weeks. (F) Heart rate and (G) ejection fraction in wild-type and *Dmd Δ52-54* mice was analyzed at 12, 28 and 52 weeks using echocardiography. In B-D and F-G, *n*=5-6 for WT, *n*=5-7 for *52-54*. All data are presented as the mean±s.d. Statistical analyses were performed using Student's *t*-test. ns, not significant; *P<0.05; **P<0.01.

Loss of dystrophin negatively impacts the heart function of DMD patients and can lead to progressive hypertrophy, arrhythmia or dilation of the heart (Hermans et al., 2010). Although heart weight of *Dmd Δ52-54* mice did not consistently indicate larger hearts throughout the 12-, 28- and 52-week time points (Fig. S1A), *Dmd Δ52-54* cardiomyocytes had an increased cell surface area of ∼1.4-fold at both 12 and 52 weeks compared to those of wild-type mice (Fig. 6A). Cardiac hypertrophy in *Dmd Δ52-54* mice was further validated using echocardiography (Fig. S1B), which was observed throughout 12 to 52 weeks of age. During systole and diastole, there was a 15-25% and 8-21% increase in posterior wall thickness, respectively (Fig. 6B; Fig. S1C). This was coupled with a reduction in chamber systolic volume by 41% and diastolic volume by 22% (Fig. 6C; Fig. S1D). In addition, ventricular diameter was reduced by 19% during systole and 9% during diastole, collectively indicating a constrictive hypertrophic response (Fig. 6D; Fig. S1E). To assess cardiac stress, we examined the expression levels of *Nppa* which encodes the atrial natriuretic peptide (ANP). *Nppa* expression increased by 2.6-fold in *Dmd Δ52-54* mice compared to expression in wild-type mice at 12 weeks, indicating a stress response (Fig. 6E). Similarly, heart rate was significantly increased by 22%, 17% and 27% during 12-, 28- and 52-week time points, indicating consistent tachycardia in *Dmd Δ52-54* mice (Fig. 6F). The ejection fraction and fractional shortening were also increased in *Dmd Δ52-54* mice by 22% and 36% respectively (Fig. 6G; Fig. S1F). Overall, *Dmd Δ52-54* mice present with cardiac hypertrophy and tachycardia throughout their lifespan, recapitulating the heart phenotype of DMD patients.

## DISCUSSION

As the development of therapies for DMD advances, the demand for representative DMD animal models also grows to evaluate their efficacies and long-term impact. Thus far, pre-clinical research studying strategies involving restoration of a truncated but functional version of dystrophin has been conducted most frequently in the *mdx* model, which is the most commonly used dystrophin-deficient mouse model (Liu et al., 2005; Long et al., 2016; Mann et al., 2001; Nelson et al., 2016; Tabebordbar et al., 2016). The *mdx* mouse strain carries a point mutation in exon 23 of *Dmd*, resulting in a premature stop codon, and therefore lacks dystrophin expression (Sicinski et al., 1989). While *mdx* is the most studied mouse model for DMD, it does not fully mimic human DMD progression. *Mdx* mice have an early onset of muscle degeneration and necrosis at 3 weeks, followed by rapid muscle regeneration in skeletal muscles that compensates for the previous myonecrosis by 6 weeks (Duddy et al., 2015). Although *mdx* mice only exhibit mild muscle degeneration in the limb muscle, the disease progression in the diaphragm is more representative of what is seen in patients with DMD (Louboutin et al., 1993; Stedman et al., 1991). However, DMD patient cardiomyopathy, which is detected during childhood, is not reflected in the *mdx* mice. Although *mdx* mice exhibit an elevated heart rate at 10-12 weeks, cardiac fibrosis and ventricular hypertrophy are not observed until 29 and 42 weeks of age, respectively (Au et al., 2011; Chu et al., 2002; Quinlan et al., 2004).

This mild cardiac phenotype in *mdx* mice is attributed to the elevated expression of utrophin, which is an autosomal homolog of dystrophin (Love et al., 1989; Matsumura et al., 1992). Utrophin has 80% sequence similarity to the C-terminal end of dystrophin and also binds to the DGC, and therefore can functionally compensate for a lack of dystrophin (Deconinck et al., 1997). Although utrophin is expressed at the sarcolemma in prenatal stages, a 4-fold increase in utrophin levels have been observed in the *mdx* adult heart (Matsumura et al., 1992). The functionality of utrophin in *mdx* mice was further emphasized when *mdx* mice were crossed to utrophin knockout mice, generating the *mdx*:*utrn^−/−^* mouse strain (Grady et al., 1997). The *mdx*:*utrn^−/−^* strain exhibits muscle deterioration starting at 2 weeks, with evident fibrotic development by 10 weeks of age (Grady et al., 1997). Although *mdx:utrn^−/−^* mice show degeneration in skeletal and cardiac muscle, worsened cardiac contractility, increased heart rate and a stunted life expectancy like DMD patients, cardiac hypertrophy and ventricular dilation are not reflected in this strain (Grady et al., 1997; Janssen et al., 2005).

In an effort to represent DMD patient disease manifestation in a mouse model, the *mdx* model was backcrossed into the DBA/2J genetic background, producing the D2-*mdx* mouse line, which exhibits severe muscular dystrophy (Fukada et al., 2010). Unlike the original *mdx* strain on the C57BL/10 background, elevated atrophy, fibrosis and calcification occurs in the skeletal muscles of D2-*mdx* mice, which correlates with weaker muscle strength (Coley et al., 2016; van Putten et al., 2019). In the heart, fibrosis and calcification are already apparent in D2-*mdx* mice at 10 weeks, and cardiac hypertrophy was observed at 34 weeks. Although cardiac stress is detected in D2-*mdx* (van Putten et al., 2019), there is no significant difference in heart rate or systolic and diastolic function compared to that of DBA/2J wild-type mice (Hakim et al., 2017). Therefore cardiac fibrosis found in D2-*mdx* at 25 weeks of age is most likely attributable to the DBA/2J background (Hakim et al., 2017). Also, calcification observed in the skeletal and heart muscles is uncommonly found in DMD patients, further emphasizing the limitations of using D2-*mdx* mice to understand the full systemic impact of DMD-targeting therapies (Hakim et al., 2017; Verhaart et al., 2019).

Several mouse models carrying DMD patient-specific deletion mutations have been generated using CRISPR/Cas9. Deletion of *DMD* exon 45 is the most common *DMD* exonic deletion mutation observed in patients, and the mutation has been introduced into the humanized DMD (hDMD) mouse strain, where the human *DMD* gene is inserted into the mouse chromosome 5 (Young et al., 2017). The resultant strain was then crossed to D2-*mdx* mice, generating hDMD del45 mdxD2 mice (Young et al., 2017). Although the hDMD del45 mdxD2 strain shows dystrophic histology such as inflammation, calcium deposits and fibrosis, skeletal and cardiac function has not been assessed (Young et al., 2017). Single exon deletion of *DMD* exon 50 (ΔEx50) and exon 44 (ΔEx44) are also among the most commonly reported DMD deletion mutations (Bladen et al., 2015). These mutations have been introduced into the endogenous mouse *Dmd* gene on a C57BL/6J background, and both the resulting mouse strains exhibit severe muscular dystrophy and weakened muscle strength, but cardiac function has not been addressed (Amoasii et al., 2017; Min et al., 2019). The largest patient *DMD* deletion recapitulated in a mouse model is the deletion of *Dmd* exons 8-34 (del8-34) (Egorova et al., 2019). Del8-34 mice show histological and muscle function phenotypes of muscular dystrophy, but the cardiac function was also not evaluated (Egorova et al., 2019). Taken together, these studies illustrate the void in mouse models recapitulating the DMD disease progression in both skeletal and heart muscles.

Here, we generated the *Dmd Δ52-54* model, which faithfully emulates the DMD patient disease phenotype. *Dmd Δ52-54* mice lack dystrophin expression, and DGC components essential to muscle function and structure are not recruited to the sarcolemma. Therefore *Dmd Δ52-54* mice experience muscle deterioration and fibrotic development, leading to compromised muscle strength, which is observed from 12 weeks to 52 weeks. The *mdx* diaphragm and *Dmd* *Δ52-54* diaphragm follow similar DMD progression and have comparable levels of centralized nuclei and fibrosis (Giordano et al., 2015). However, *Dmd Δ52-54* mice have an early onset of cardiac dysfunction at 12 weeks, unlike the *mdx* and D2-*mdx* models used in pre-clinical studies. This cardiac phenotype includes ventricular hypertrophy, reduced ventricular volume and tachycardia that persists throughout 12 to 52 weeks. In DMD patients, the ejection fraction declines as the disease progresses, reaching levels as low as 25-30% (Meyers and Townsend, 2019; Tandon et al., 2015). This decrease in ejection fraction is not recapitulated in the *Dmd Δ52-54* mice. The elevated ejection fraction and fraction shortening in the *Dmd Δ52-54* mice are likely due to increased sympathetic activity in DMD, which was also previously reported in the *mdx* mouse model, consequently causing increased heart rate and contractility (Chu et al., 2002; Puhl et al., 2016). Although DMD patients mainly present with dilated cardiomyopathy, *Dmd Δ52-54* mice exhibit a phenotype similar to HCM. However, left ventricular remodeling has been observed in ∼50% of end-stage patients with HCM, transitioning into DCM (Harris et al., 2006). Therefore, the *Dmd Δ52-54* model should be monitored to later stages to understand whether the hypertrophic cardiac phenotype will progress into DCM. Overall, *Dmd Δ52-54* is a representative model of DMD disease progression, and its early disease manifestation allows it to be a powerful tool in understanding long-term therapeutic outcomes impacting both skeletal and cardiac muscles.

Because the *Dmd Δ52-54* deletion disrupts the ORF, the mutation is amenable to single exon or multi-exon skipping to restore the reading frame while converting the out-of-frame deletion into an in-frame deletion representative of BMD. Exon 55 can be excluded in the *Dmd Δ52-54* model, creating a truncated dystrophin protein without affecting dystrophin functional domains – a change that is applicable to 2.7% of all DMD patients carrying deletion mutations (Aartsma-Rus et al., 2009). Multi-exon removal can also be studied using the *Dmd Δ52-54* model, creating a shorter dystrophin protein without exons 45-55. Since patients carrying the deletion of *DMD* exons 45-55 are asymptomatic or present with mild BMD, strategies yielding this mutation could impact 63% of all DMD patients with deletion mutations (Béroud et al., 2007). Restoring the ORF by excluding *DMD* exons can be executed through antisense oligonucleotide (ASO) treatment or gene editing (Aartsma-Rus et al., 2009; Wong and Cohn, 2017). To date, skipping of *DMD* exons 45-55 using ASO treatment has been studied in patient cells, *mdx52* mice (which carry a *Dmd* exon 52 deletion) and a humanized DMD mouse model to understand the skipping efficiency and/or level of dystrophin restoration (Aoki et al., 2012; Echigoya et al., 2015, 2019; Lee et al., 2018; van Vliet et al., 2008). However, improvement in cardiac function was not assessed in these studies, because cardiac pathology has not been described in these mouse models.

Considering the progressive DMD disease observed in *Dmd Δ52-54* mice, this model could be utilized to study mutation-dependent strategies through ASO treatment or gene editing. Moreover, *Dmd Δ52-54* mice could be utilized as an informative dystrophin-deficient model for mutation-independent strategies, such as upregulation of utrophin or other DMD disease modifiers (van den Bergen et al., 2015; Vo and McNally, 2015; Weir et al., 2004; Wojtal et al., 2016).

In summary, we generated the first mouse model carrying a *Dmd* multi-exonic deletion, derived from a 116 kb deletion within the *Dmd* mutational hot spot. Comprehensive characterization of *Dmd Δ52-54* mice revealed a dystrophic phenotype in skeletal muscles leading to locomotor dysfunction. To our knowledge, *Dmd Δ52-54* is the first mouse model carrying a *Dmd* deletion mutation to exhibit early onset of a dystrophic cardiac phenotype and cardiac functional abnormalities, as shown through fibrotic development, ventricular hypertrophy and tachycardia. Therefore, due to its early skeletal and cardiac phenotype, the *Dmd Δ52-54* mouse strain represents an animal model for DMD pre-clinical studies, where long term therapeutic implications can be thoroughly evaluated.

## MATERIALS AND METHODS

### sgRNA design

sgRNAs specific for *Streptococcus pyogenes* Cas9 were designed for the generation of the *Dmd Δ52-54* mouse line using the benchling.com online tool (Table 1).

**Table 1.**
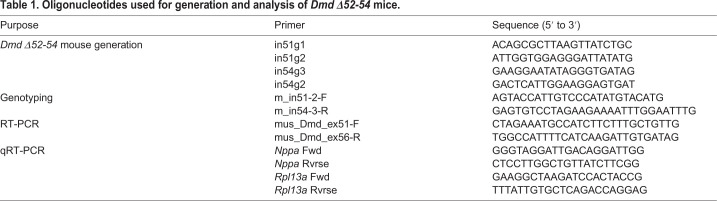
**Oligonucleotides used for generation and analysis of *Dmd Δ52-54* mice.**

### Synthesis of sgRNAs and Cas9 mRNA

All single guide (sgRNAs) were synthesized as described by Gertsenstein and Nutter (2018). Injection mixes for pronuclear injections included 10 ng/µl of each sgRNA and 20 ng/µl of Cas9 mRNA (Life Technologies; A23978) (Gertsenstein and Nutter, 2018).

### Pronuclear microinjections

For embryo donors, we used 3- to 4-week-old C57BL/6J (Jackson) females. Pseudopregnant surrogates were CD-1 (ICR) females (Charles River).

All procedures were conducted according to Behringer et al., 2014. In brief, embryo donors were super-ovulated and mated overnight with male breeders. Successfully mated females were selected and the oviducts were dissected to isolate fertilized embryos. The embryos were subjected to pronuclear microinjection with sgRNAs and Cas9 mRNA, and transferred into the oviducts of surrogate females (Behringer et al., 2014). Mice were backcrossed for three generations and then intercrossed before being used for analysis. Only male mice were used in this study. All animal procedures were conducted in compliance with the Animals for Research Act of Ontario and the Guidelines of the Canadian Council on Animal Care. Animal protocols performed at The Centre for Phenogenomics (TCP), Toronto, were reviewed and approved by the local Animal Care Committee.

### DNA and RNA extraction and analyses

For genotyping analysis, mouse tails were biopsied at 2 weeks old, and DNA was extracted using a DNAeasy Blood and Tissue kit (Qiagen). One microliter of the yielded DNA was used for PCR with DreamTaq polymerase (Thermo Fisher Scientific) and the primers shown in Table 1. For in-depth molecular analyses, DNA from tissues was isolated using a DNAeasy Blood and Tissue kit (Qiagen). For RNA isolation, mouse tissues were sectioned, collected in 1.4-mm Zirconium Bead pre-filled tubes (OPS Diagnostics) and homogenized using a MagNA Lyser (Roche Diagostic). RNA was then extracted using TRIzol Reagent (Thermo Fisher Scientific).

One microgram of RNA was used for cDNA synthesis by SuperScript III reverse transcriptase (Thermo Fisher Scientific). The cDNA was used in subsequent RT-PCR with the primers shown in Table 1. For qRT-PCR, 5 ng of cDNA was used for every reaction, and reactions were performed using WISENT ADVANCED qPCR mastermix with SUPERGREEN dye (Wisent) on a CFX384 Touch real-time PCR Detection System (Bio-Rad) (primers in Table 1). All samples were run in triplicate. Data were analyzed using CFX Manager Software (Bio-Rad) and normalized to *Rpl13a* expression levels.

### Whole genome sequencing

DNA extracted from *Dmd 52-54* mouse tails was utilized for whole genome sequencing (WGS), which was performed using the Illumina HiSeq X system (San Diego, CA, USA) by The Centre for Applied Genomics (TCAG) at the Hospital for Sick Children. A Qubit Fluorometer High Sensitivity Assay (Thermo Fisher Scientific, Waltham, MA, USA) was used to evaluate DNA yield and the Nanodrop (Thermo Fisher Scientific) OD260/OD280 ratio was used to check DNA purity. In brief, 400 ng of each DNA sample was used for library preparation using the Illumina TruSeq PCR-free DNA Library Prep Kit, where DNA was sonicated into an average of 350-bp fragments. A-tailed and indexed TruSeq Illumina adapters were ligated to end-repaired sheared DNA fragments before the library was amplified. Libraries were analyzed using Bioanalyzer DNA High Sensitivity chips (Agilent Technologies, Santa Clara, CA, USA) and quantified using qPCR. The libraries were loaded in equimolar quantities and pair-end sequenced on the Illumina HiSeqX platform to generate 150-bp reads. Integrative Genomics Viewer (IGV) version 2.8.2 was used for analysis with GRCm38/mm10 as the murine reference genome. The top ten predicted off-target sites were identified using benchling.com.

### Functional tests

Forelimb and hindlimb grip strength tests were performed by The Centre for Phenogenomics in Toronto based on the TREAT-NMD SOP DMD_M.2.2.001 protocol. Age matched 12-, 28- and 52-week-old male C57BL/6J and *Dmd Δ52-54* were lowered over the grid of the grip strength meter (Bioseb) with the torso parallel to the grid. Forepaws and hindpaws were allowed to attach to the grid before pulling the mouse back by the tail, and the maximal grip strength value of the mouse was recorded. The test was done in triplicates, where the average grip strength value was corrected by the mouse body weight.

For the echocardiography, male mice were scanned using the Vevo2100 ultrasound machine (VisualSonics, Toronto, Canada) with a 30 MHz transducer as described previously (Zhou et al., 2005). All mice were scanned under 1.5% isoflurane anesthesia for 20-30 min with careful monitoring of the body temperature to maintain it at 37-38°C (TREAT-NMD DMD_M.2.2.003). All measurements were conducted using the cardiac package of the Vevo 2100 v1.6.0 software.

*In vivo* tibialis anterior contraction tests were performed as previously described (Kemaladewi et al., 2019). Briefly, contractile activity was measured using the 1300A: 3-in-1 Whole Animal System and analyzed using the Dynamic Muscle Analysis 5.5 and 5.3 high-throughput software (Aurora Scientific). The mice were anaesthetized using ketamine-xylazine solution at 100 mg/kg and 10 µl mg/kg to body weight, respectively, through intraperitoneal injection. Percutaneous electrodes were placed in the tibialis anterior and contractile output was measured.

### Clinical chemistry

Mice were euthanized using cervical dislocation and whole blood was collected into BD Microtainer Capillary Blood Collector tubes (Fisher Scientific) from the chest cavity immediately after heart dissection. Blood was centrifuged at 10,000 ***g*** at 4°C for 5 min. Clear serum was extracted and stored at −80°C.

Serum was measured using the Liquid Creatine Kinase Reagent Kit (Pointe Scientific) as per the manufacturer's protocol. In brief, serum was diluted in 1× PBS at a 1:4 ratio and incubated with reagent for 2 min. Absorbance was measured at 340 nm and readings were recorded every 2 min two more times. Final serum creatine kinase (CK) was calculated using the manufacturer's protocol, and serum CK was plotted relative to an average of serum CK levels of seven (12 weeks) and four wild-type mice (52 weeks).

### Tissue processing

Quickly after cervical dislocation, mouse hearts were arrested in diastole through direct KCl (1 M KCl in PBS) injection. All muscles dissected were frozen in cooled isopentane in liquid nitrogen as previously described (Nelson et al., 2016).

### Histological staining

All muscles were sectioned at 8 µm for histological staining. Hematoxylin and Eosin (H&E) staining was conducted using a standard protocol (Fischer et al., 2008). H&E slides were then scanned using the 3DH Pannoramic Slide Scanner by the Imaging Facility at the Hospital for Sick Children. CaseViewer (3DHISTECH) was used for image acquisition. Centralized nuclei were quantified using ImageJ 1.52a software from a total of 300 myofibers.

Trichrome staining was performed at the Pathology lab at The Centre for Phenogenomics, Toronto (TREAT-NMD SOP MDC1A_M.1.2.003). Trichrome-stained slides were scanned using a Hamamatsu Nanozoomer and analyzed using the NDP.view2 Viewing Software. Three frames containing at least 300 fibers each were used for fibrosis quantification using ImageJ 1.51 software.

### Immunofluorescence staining and analysis

All muscle tissues were sectioned at 8 µm for immunofluorescence staining. Sections were fixed in ice-cold methanol and blocked with blocking buffer (3% normal goat serum and 0.2% BSA in PBS). Primary antibodies were incubated overnight at 4°C. Primary antibodies used were rabbit polyclonal anti-dystrophin (abcam15277; Abcam; 1:200), rabbit polyclonal anti-syntrophin-α 1 (ab11187; Abcam; 1:600), rabbit polyclonal anti-β-sarcoglycan (ab222241; Abcam; 1:100) and rat monoclonal anti-laminin-2 (α2 chain; 4H8-2; Sigma Aldrich; 1:500). Secondary antibodies used were goat polyclonal anti-rabbit Alexa Fluor 594-conjugated (Thermo Fisher Scientific; 1:250) and goat polyclonal anti-rat Alexa Fluor 488-conjugated (Thermo Fisher Scientific; 1:250) antibodies.

For wheat germ agglutinin (WGA) staining, sections were incubated with 10 µg/ml wheat germ agglutinin conjugated to Alexa Fluor 594 (W11262; Invitrogen). Cell surface area was quantified from at least 100 cardiomyocytes using ImageJ 1.51 software.

For sections stained with anti-laminin-α2, slides were scanned as described previously (Kemaladewi et al., 2017) and Feret's diameter was quantified (TREAT-NMD SOP DMD-M1.2.001) using Open-CSAM in the ImageJ 1.51 software (Desgeorges et al., 2019).

### Western blotting

Protein was extracted from homogenized mouse tissue by adding a 1:1 solution of RIPA homogenizing buffer (50 mM Tris-HCl pH 7.4, 150 mM NaCl and 1 mM EDTA) and RIPA double-detergent buffer (2% deoxycholate, 2% NP-40, 2% Triton X-100 in RIPA homogenizing buffer) supplemented with protease inhibitor cocktail (Roche), as previously described (Kemaladewi et al., 2019). Total protein concentration was quantified using a Pierce BCA protein assay kit (Thermo Fisher Scientific). Protein (15 μg) was prepared and western blotting was conducted according to the NuPAGE electrophoresis system (Thermo Fisher Scientific). Primary antibodies utilized were mouse monoclonal anti-dystrophin (MANDYS8; Sigma Aldrich; 1:5000), mouse monoclonal anti-vinculin (V284; Millipore; 1:2500) and mouse monoclonal anti-β-actin (sc-47778; Santa Cruz Biotechnology; 1:10,000).

### Statistical analysis

GraphPad Prism version 7 was used to conduct Student's *t*-tests for all statistical analyses.

## Supplementary Material

Supplementary information
