# Correction: A novel mouse model of Duchenne muscular dystrophy carrying a multi-exonic *Dmd* deletion exhibits progressive muscular dystrophy and early-onset cardiomyopathy

**DOI:** 10.1242/dmm.052372

**Published:** 2025-04-04

**Authors:** Tatianna Wai, Ying Wong, Abdalla Ahmed, Grace Yang, Eleonora Maino, Sydney Steiman, Elzbieta Hyatt, Parry Chan, Kyle Lindsay, Nicole Wong, Diane Golebiowski, Joel Schneider, Paul Delgado-Olguín, Evgueni A. Ivakine, Ronald D. Cohn

There was an error in *Dis. Model. Mech.* (2020) **13**, dmm045369 (doi:10.1242/dmm.045369).

The wild-type (WT) triceps image in Fig. 4G was inadvertently duplicated and shown again as the WT gastrocnemius image. This figure has been amended to show WT gastrocnemius histological staining and the corrected panel is shown below.

**Fig. 4G:**
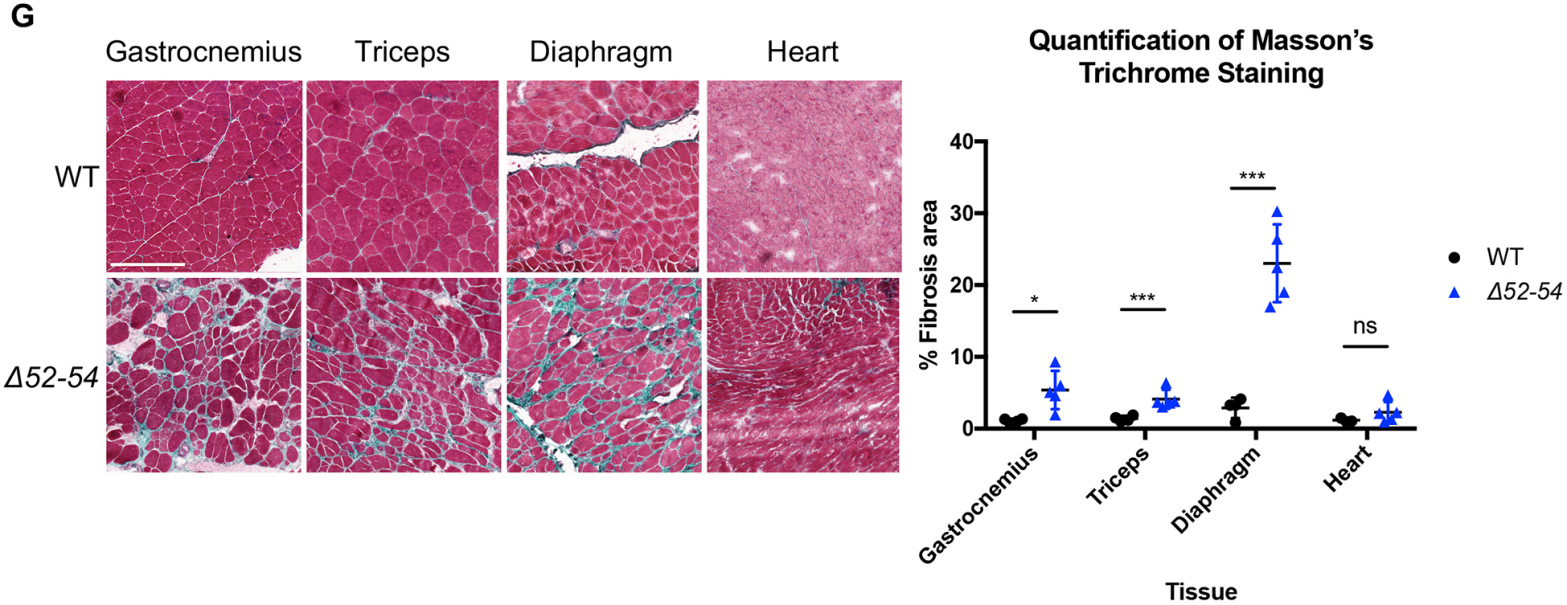
**(corrected panel). *Dmd Δ52-54* mice exhibit dystrophic histology at 52 weeks.** (G) Masson's trichrome staining was performed on gastrocnemius, triceps, diaphragm and heart tissues of 52-week-old wild-type (*n*=3-4) and *Dmd Δ52-54* (*n*=5) mice, and the fibrotic area was quantified. Scale bar: 250 μm. All data are presented as the mean±s.d. Statistical analyses were performed using a Student's *t*-test. ns, not significant; *P<0.05; **P<0.01; ***P<0.001; ****P<0.0001.

The authors apologise for this error and any inconvenience it may have caused.

