# Supplementary material for: A novel mouse model of Duchenne muscular dystrophy carrying a multi-exonic Dmd deletion exhibits progressive muscular dystrophy and early-onset cardiomyopathy
Source: Dis Model Mech. 2020 Sep 21;13(9):dmm045369. doi: 10.1242/dmm.045369 (PMC7522028; doi:10.1242/dmm.045369)
Supplement: Supplementary information [file dmm-13-045369-s1.pdf]

Supplementary Figures

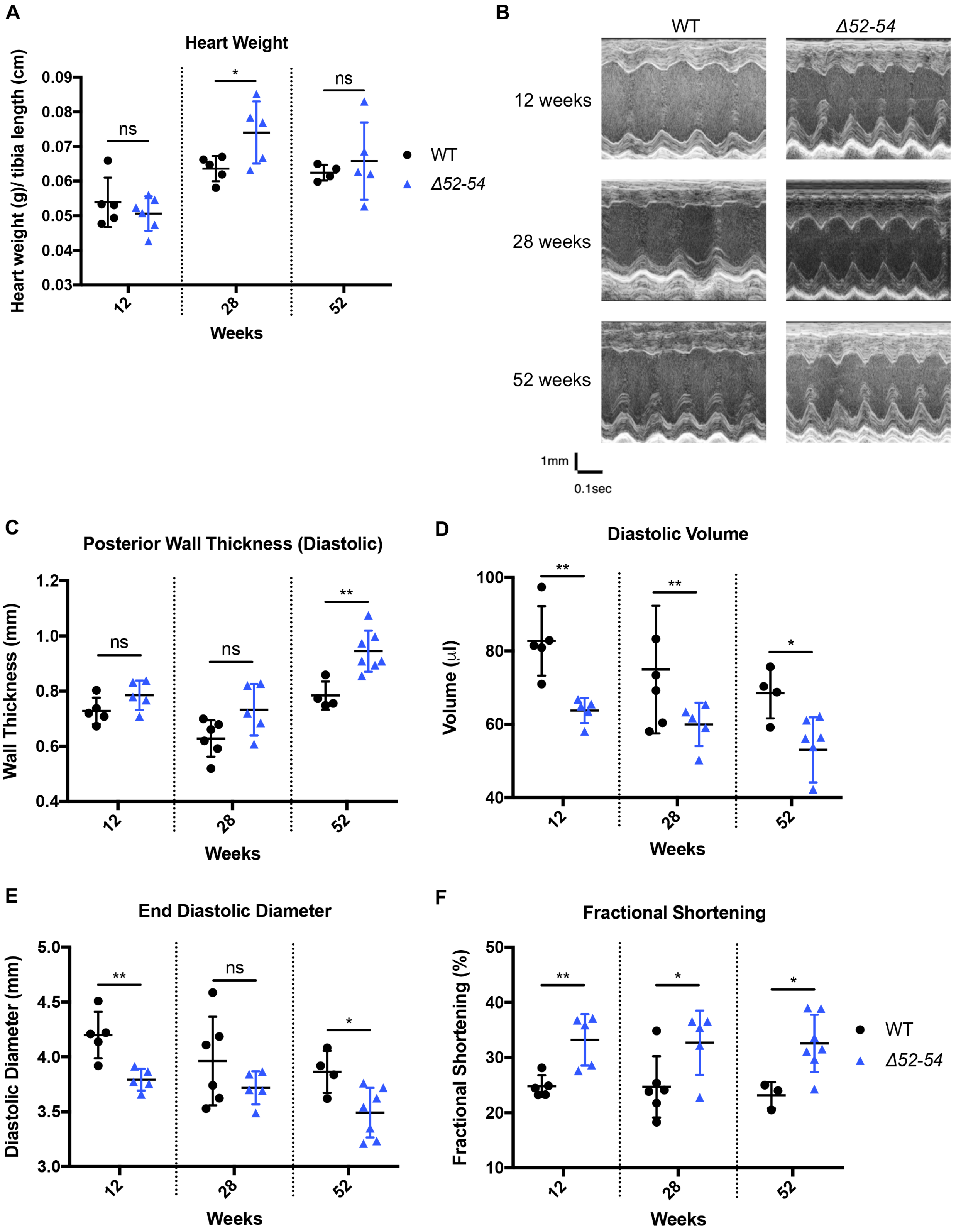

Figure S1. ***Dmd Δ52-54* present cardiac hypertrophy while the heart in *Dmd Δ52-55* are unaffected.** (A) Heart weight of wildtype and *Dmd Δ52-54* mice at 12, 28, and 52 weeks were normalized to respective tibia lengths. (B) Representative M-mode tracing of the left ventricle in wildtype and *Dmd Δ52-54* mice at 12, 28, and 52 weeks. The heart in wildtype (WT) and *Dmd Δ52-54* (*Δ52-54*) mice were analyzed using echocardiography to determine the diastolic measurements (C) left ventricular posterior wall thickness, (D) left ventricular volume, and (E) left ventricular end diameter, as well as (F) fractional shortening. All data is represented as the mean ± S.D. Statistical analyses were performed with Student's t-test. *P < 0.05, ** P < 0.01.
